# Non-canonical autophagy LAPs lupus

**DOI:** 10.1038/cdd.2016.55

**Published:** 2016-06-10

**Authors:** A K Simon, A J Clarke

**Affiliations:** 1 Kennedy Institute of Rheumatology, University of Oxford, Roosevelt Drive, Headington, Oxford, UK; 2 MRC Human Immunology Unit, Weatherall Institute of Molecular Medicine, University of Oxford, John Radcliffe Hospital, Headley Way, Oxford, UK

Systemic lupus erythematosus (SLE) is an autoimmune disease whose central pathology is the recognition of, and reaction against, nuclear self-antigens by the immune system. This leads to type I interferon signalling, and the production of pathogenic auto-antibodies, which mediates much of the disease. The clinical consequences of the autoimmune response can be severe – common manifestations include rash, renal inflammation progressing to kidney failure, and neuropsychiatric involvement. The prevalence of SLE is approximately 0.1% – but why is it not much higher, given that many billions of cells die each day as part of normal tissue homeostasis? In the 5th May issue of *Nature*, Martinez *et al.*^1^ provide a new insight into how these apoptotic cell corpses are removed before they can incite an inflammatory response.

Phagocytosis of apoptotic cells is performed by macrophages and immature dendritic cells, and it is known that genetic variants affecting components of this process (e.g., ITGAM^2^) can predispose to SLE. Similarly, deletion of many of the genes required for phagocytosis of apoptotic cells leads to a lupus-like disease in mice.^3^ Genome-wide association studies have consistently highlighted autophagy genes as risk loci in lupus,^4^ and Martinez *et al.* now show that one potential mechanism for this link is that a non-canonical form of autophagy, known as LC3-associated phagocytosis (LAP), is required for effective clearance of apoptotic cells (Figure 1).

Canonical macroautophagy starts with the assembly of a pre-initiation complex consisting of ULK1, FIP200, and ATG13, which in turn leads to activation of the VPS34/Beclin-1 PI3K complex, and then formation and extension of a double-membraned autophagosome around cellular contents by the lipidation of LC3, through the action of two ubiquitin-like conjugation systems. ULK1 is subject to regulatory phosphorylation by mTOR and AMPK, and this provides a means for the control of autophagy in response to nutrient status.

LAP, however, differs from canonical autophagy in a number of important ways. LAP is activated by pattern recognition receptors (e.g., TLR4) during phagocytosis. There is rapid recruitment of a subset of the autophagy machinery to the phagosome, which becomes decorated with LC3 and rapidly trafficked to and degraded by the lysosome. Unlike canonical autophagy in which a double-membraned autophagosome forms, the LC3-associated phagosome has only a single layer, and LAP also occurs much more quickly, with LC3 association occurring within minutes rather than hours.^5^ Although LAP shares the VPS34/Beclin-1 PI3K complex, ULK1 is not required. However, the protein Rubicon, which associates with the UVRAG-containing class III PI3K complex, and negatively regulates canonical autophagy, is essential for LAP.^6^

LAP has been shown to be important in the phagocytosis of nucleic acid immune complexes by plasmacytoid dendritic cells in lupus,^7^ and *ex vivo* in macrophages for the clearance of intracellular pathogens and dead cells.^5, 8^

Martinez *et al.* conditionally deleted genes involved in initiation of canonical autophagy, those specific to LAP, and also genes common to both processes, using a LysM-cre system that targets macrophages, monocytes, neutrophils, and some conventional dendritic cells. They found that from approximately 6 months of age, mice with deletions in genes common to both canonical autophagy and LAP, but not those specific to canonical autophagy, developed an auto-inflammatory syndrome with many features typical of lupus, that is, type I interferon production and autoantibody formation. These mice went on to develop nephritis and kidney damage, just as in SLE. Importantly, knockout of LAP-specific genes (e.g., Rubicon) also leads to the disease. Mechanistically, they found that uptake of dying cells by macrophages was intact, but that the phagocytosed apoptotic cells were not digested, and instead elicited an inflammatory response. The lupus-like disease was further exacerbated by injections of exogenous apoptotic cells.

This study provides some important mechanistic and experimental insights. It illustrates the critical role LAP plays in digesting phagocytosed dead cells and avoiding autoimmunity *in vivo*. It therefore suggests one way in which genetic risk variants in autophagy genes may predispose to lupus. Secondly, the contribution of each subset of the autophagy machinery is dissected using a comprehensive panel of deletion models. The overwhelming majority of studies examining the role of autophagy in a biological process use only deletion of a single autophagy gene. Although this is clearly an informative and valid experimental approach, this work does illustrate that mechanistic subtlety may be overlooked. A further example is from Kimmey *et al.,*^9^ who show that conditional deletion of *Atg5*, also using LysM-cre, leads to dramatically impaired immunity against *Mycobacterium tuberculosis* infection. However, deletion of other autophagy genes, including *Ulk1*, *Atg7*, *Atg16l1*, and *Atg3*, did not affect survival with this pathogen, indicating an autophagy-independent role for *Atg5*.

An interesting point is to what extent the loss of autophagy, either LAP or canonical, affects the effector immune response in their models. Macrophages are key players in the development of immune-complex-mediated glomerulonephritis,^10^ activated through their Fc receptors by immune complexes deposited in the glomerulus. Recent work has shown that canonical autophagy genes play an anti-inflammatory role in myeloid cells, limiting sterile lung inflammation.^11^ Whether this function may also apply in the response to immune complexes in the kidney is an open question.

Finally, the major question presented by this work is to what extent LAP contributes to the pathogenesis of human SLE. It would be interesting to see to what extent disease-associated genetic variants in autophagy genes affect LAP in human cells, and to investigate pharmacological modulation of LAP as a potential therapeutic approach.

## Figures and Tables

**Figure 1:**
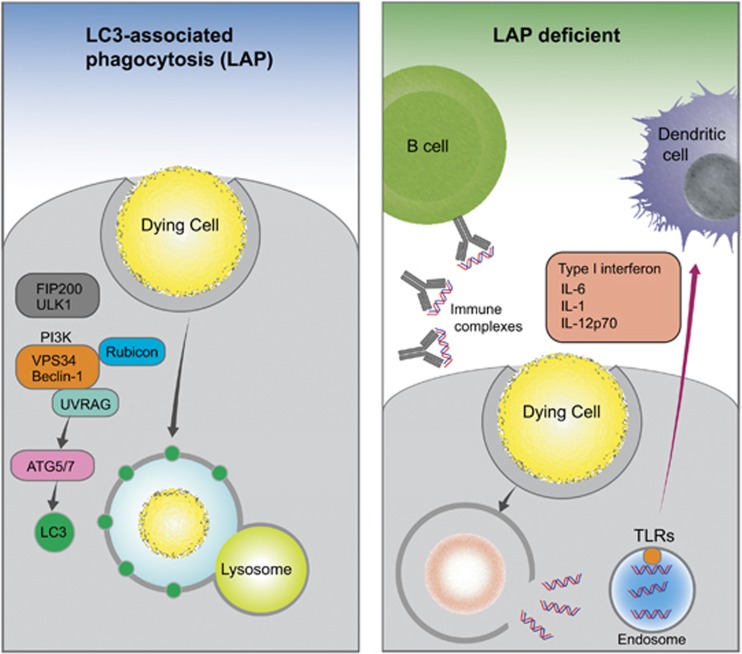
Non-canonical autophagy in the clearance of dead cells. LC3-associated phagocytosis (LAP) is the process by which phagosomes become decorated with LC3 and are subsequently trafficked to the lysosome for degradation. LAP uses a subset of the canonical autophagy machinery, but notably does not need the ULK1 pre-initiation complex, and again, distinct from canonical autophagy, requires Rubicon and Nox2. Following ingestion, dying cells are rapidly degraded and do not induce an autoimmune response. However, when components of the LAP pathway are genetically deleted, although dying cells are engulfed normally they are not degraded and hence the nuclear material escapes and leads to an immune response. Consequently, a systemic inflammatory disease resembling lupus develops

## References

[bib1] Martinez J et al. Nature 2016; 533: 115–119. 27096368 10.1038/nature17950 PMC4860026

[bib2] Rhodes B et al. Ann Rheum Dis 2012; 71: 2028–2034. 22586164 10.1136/annrheumdis-2012-201390 PMC3488763

[bib3] Green DR, Oguin TH, Martinez J. Cell Death Differ 2016; 23: 915–926. 26990661 10.1038/cdd.2015.172 PMC4987729

[bib4] Bentham J et al. Nat Genet 2015; 47: 1457–1464. 26502338 10.1038/ng.3434 PMC4668589

[bib5] Martinez J et al. Proc Natl Acad Sci USA 2011; 108: 17396–17401. 21969579 10.1073/pnas.1113421108 PMC3198353

[bib6] Martinez J et al. Nat Cell Biol 2015; 17: 893–906. 26098576 10.1038/ncb3192 PMC4612372

[bib7] Henault J et al. Immunity 2012; 37: 986–997. 23219390 10.1016/j.immuni.2012.09.014 PMC3786711

[bib8] Sanjuan MA et al. Nature 2007; 450: 1253–1257. 18097414 10.1038/nature06421

[bib9] Kimmey JM et al. Nature 2015; 528: 565–569. 26649827 10.1038/nature16451 PMC4842313

[bib10] Mohan C, Putterman C. Nat Rev Nephrol 2015; 11: 329–341. 25825084 10.1038/nrneph.2015.33

[bib11] Lu Q et al. Cell Host Microbe 2016; 19: 102–113. 26764600 10.1016/j.chom.2015.12.011 PMC4714358

